# Rosmarinic Acid Ameliorates *Dermatophagoides farinae* Extract-Induced Atopic Dermatitis-like Skin Inflammation by Activating the Nrf2/HO-1 Signaling Pathway

**DOI:** 10.3390/ijms252312737

**Published:** 2024-11-27

**Authors:** Ki-Shuk Shim, Hye Jin Kim, Kon-Young Ji, Dong Ho Jung, Sun Haeng Park, Hyun-Kyung Song, Taesoo Kim, Ki Mo Kim

**Affiliations:** 1 KM Convergence Research Division, Korea Institute of Oriental Medicine, Yuseong-daero 1672, Yuseong-gu, Daejeon 34054, Republic of Korea; angeloshim@kiom.re.kr (K.-S.S.); kimhyejin43@kiom.re.kr (H.J.K.); jky8387@kiom.re.kr (K.-Y.J.); jdh9636@kiom.re.kr (D.H.J.); sun7710@kiom.re.kr (S.H.P.); 2 Practical Research Division, Honam National Institute of Biological Resources, Gohadoan-gil 99, Mokpo 58762, Republic of Korea; gusrud1654@hnibr.re.kr

**Keywords:** rosmarinic acid, atopic dermatitis, Nrf2, HO-1, keratinocytes

## Abstract

Atopic dermatitis (AD) is one of the most common chronic inflammatory skin diseases. AD pathogenesis is associated with increased oxidative stress, impairment of the skin barrier, and activation of the immune response. Rosmarinic acid (RA), a caffeic acid ester, is known for its anti-inflammatory and antioxidant properties. However, the effects of RA on *Dermatophagoides farinae* extract (DfE)-induced AD-like skin inflammation, as well as its ability to regulate oxidative stress through the Nrf2/HO-1 pathway in TNF-α/IFN-γ-treated keratinocytes, remain unclear. We investigated RA activity in a DfE-induced AD-like skin inflammation mouse model and IFN-γ/TNF-α-stimulated keratinocytes. We found that RA attenuates DfE-induced inflammation by decreasing dermatitis scores and serum inflammatory marker levels and mast cell infiltration. Additionally, RA significantly suppressed IFN-γ/TNF-α-induced chemokine production in keratinocytes and reduced Th cytokine levels in concanavalin A-stimulated splenocytes. Importantly, RA also increased Nrf2/HO-1 expression in TNF-α/IFN-γ-treated keratinocytes. In conclusion, this study demonstrated that RA effectively alleviates DfE-induced AD-like skin lesions by reducing the levels of inflammatory cytokines and chemokines. Furthermore, RA promotes Nrf2/HO-1 signaling in keratinocytes, which may help mitigate DfE-induced oxidative stress, thereby alleviating AD-like skin inflammation. These findings highlight the potential of RA as a therapeutic agent for treating AD and other skin inflammation.

## 1. Introduction

Atopic dermatitis (AD) is one of the most common chronic inflammatory skin diseases. AD pathogenesis involves allergic sensitization, oxidative stress induced by increased reactive oxygen species (ROS) generation, impaired skin barrier function, and increased Th immune reactions [1,2]. In the early response to external allergens, high amounts of ROS are rapidly generated in skin cells such as keratinocytes [3], causing the oxidization of cellular components and the degradation of extracellular matrix (ECM) components [4], which in turn stimulates immune reactions in skin lesions. Allergens activate immune cells and/or keratinocytes to produce proinflammatory cytokines and chemokines, which further evoke a systemic immune response with a predominant Th2 immune response exacerbated by mixed activation of Th1/2 cells at chronic stages of skin inflammatory diseases, such as AD [5,6]. Given the importance of oxidative stress in stimulating skin inflammation, the nuclear factor erythroid 2-related factor 2 (Nrf2)/heme oxygenase-1 (HO-1) signaling pathway has gained increasing attention [7,8]. Nrf2 is a ubiquitously expressed transcription factor that modulates redox balance to maintain skin homeostasis. Nrf2 binds to antioxidant response elements in ROS-protective genes, such as HO-1, glutathione S-transferase (GST), and superoxide dismutase (SOD) [9]. Additionally, Nrf2 promotes keratinocyte differentiation to maintain skin structure by regulating keratinocyte differentiation markers such as loricrin and keratin [10,11]. Nrf2 also protects keratinocytes from UV-induced oxidative damage [8] and reduces apoptosis in melanocytes by increasing the expression of SOD [12]. Inhibition of endogenous Nrf2 activity by a dominant-negative form of Nrf2 in loricrin-deficient epidermal cells causes severe loss of barrier function and cell death within 24 h [13]. However, excessive activation of Nrf2 in keratinocytes due to the deletion of its binding regulator, Kelch-like erythroid cell-derived protein with CNC homology-associated protein 1 (Keap1), causes epithelial abnormalities, which can be rescued by concomitant deletion of the Nrf2 gene [14]. These findings underscore the critical role of the Nrf2-dependent redox balance system in maintaining skin homeostasis.

The long-term use of AD therapeutic agents using steroids or anti-histamines has been restricted due to severe side effects [15,16]. Therefore, the research to develop promising therapeutics derived from natural resources or their components is essential and in high demand. We previously reported the ethanol extract of *Isodon inflexus* containing rosmarinic acid (RA) has therapeutic effect against DfE-induced AD-like skin inflammation in NC/Nga mice [17]. Rosmarinic acid (RA), a naturally occurring caffeic acid ester identified in *Rosmarinus officinalis* L., is known to have various pharmaceutical activities, including antioxidative activity, anti-inflammatory activity, and antitumor activity [18]. In terms of skin inflammation, RA attenuates dibutyl squarate-induced pruritus or 2,4-dinitrofluorobenzene (DNFB)-induced AD in mice by inhibiting mast cell activation or T-cell-generated inflammatory cytokine levels [19], thereby contributing to improving the skin condition of patients with AD symptoms by reducing transepidermal water loss [20]. Additionally, RA suppresses Th2-related IL-4 and IL-5 levels, reducing intratracheal DfE-induced allergic inflammation in the lung [21]. However, its effects on DfE-induced AD-like skin inflammation remain unknown. Therefore, we investigated the potential effects of RA on DfE-induced AD-like skin inflammation in an NC/Nga mouse model. Previously, RA was shown to protect against UVB-induced oxidative damage in keratinocytes by activating Nrf2/HO-1 signaling or enhancing Akt/ERK/Nrf2-regulated glutathione synthesis [22]. RA also mitigates chlorpyrifos-induced kidney injury [23], vascular calcification [24], and ischemic stroke [25] by increasing Nrf2/HO-1 signaling to reduce oxidative stress. However, its effects on inflammatory cytokine-induced Nrf2/HO-1 signaling in keratinocytes have not been investigated.

Nishiki-nezumi Cinnamon/Nagoya (NC/Nga) mice spontaneously develop skin lesions similar to those in human AD [26,27]. *Dermatophagoides farinae* extract (DfE), the most prevalent house dust mite in daily life, is a potent allergen known to trigger AD symptoms [28]. DfE-treated NC/Nga mice display major clinical skin symptoms of human AD, including dryness, erythema, edema, and excoriation [29,30].

Keratinocytes are the primary cells responsible for expressing epidermal proteins to construct skin tissue and to protect against exogeneous allergens or stresses [31]. Environmental allergens stimulate keratinocytes to release proinflammatory factors, inducing the recruitment and activation of mast cells, dendritic cells, or T-cells [32]. Thus, an in vitro culture system using keratinocytes is a valuable approach for studying the cellular and molecular aspects of epidermal impairment in AD-like inflammatory diseases.

In this study, we explored the possible inhibitory effects of RA on DfE-induced AD-like skin inflammation in an NC/Nga murine model. To elucidate the underlying molecular mechanism by which RA influences skin inflammation, we examined its effect on IFN-γ/TNF-α-induced Nrf2/HO-1 expression in human epidermal keratinocytes (HEKs). We also evaluated the impact of RA on concanavalin A (Con A)-induced inflammatory cytokine levels in mouse splenocytes and on tumor necrosis factor (TNF)-α/ interferon (IFN)-γ-induced chemokine levels in HaCaT cells.

## 2. Results

### 2.1. Ra Attenuates Dfe-Induced Skin Inflammatory Lesions, Reduced Dermatitis Scores, and Decreased the Serum Levels of Inflammatory Factors in AD Model Mice

**Figure 1:**
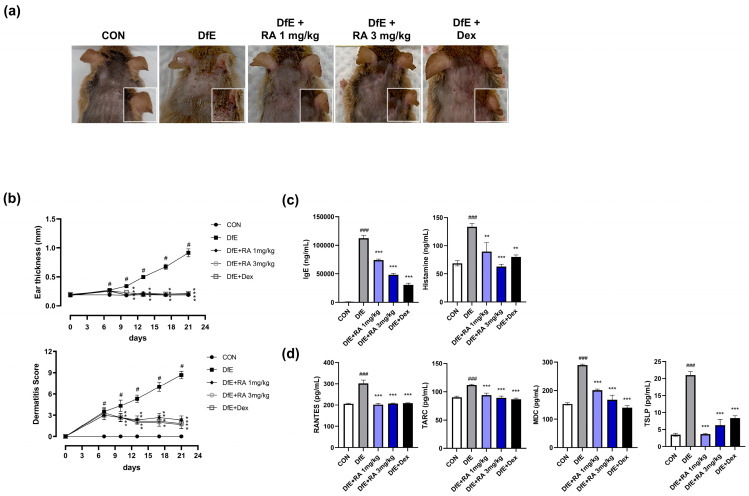
RA alleviated DfE-induced AD-like skin inflammation in NC/Nga mice. (**a**) Representative images of DfE-induced skin features in each mouse group are shown; (**b**) ear thickness and dermatitis score of dorsal lesions on each day for 21 days are presented; (**c**) serum levels of IgE, histamine, and (**d**) inflammatory chemokines (RANTES, TARC, MDC, and TSLP) in each group were examined via a bead-based immune assay. CON, normal control mice; DfE, DfE-induced mice; DfE + RA 1 mg/kg, DfE-induced RA (1 mg/kg)-administered mice; DfE + RA 3 mg/kg, DfE-induced RA (3 mg/kg)-administered mice; DfE + Dex, DfE-induced dexamethasone (1 mg/kg)-administered mice. The data are presented as the means ± standard errors of the means (SEMs) (*n* = 5). # *p* < 0.05 and ### *p* < 0.001 vs. CON; * *p* < 0.05, ** *p* < 0.01, and *** *p* < 0.001 vs. DfE.

The topical application of DfE for two weeks significantly induced the development of dryness, excoriation, hemorrhage, and hematoma in both the ear and dorsal skin lesions of mice (Figure 1a). However, skin deterioration in these lesions was notably alleviated in the mice administered with RA (1 or 3 mg/kg) or dexamethasone. This mitigation of skin lesions was supported by a gradual and significant reduction in both the dermatitis score and ear thickness in all three groups (Figure 1b). The dermatitis score in the RA-treated groups decreased to less than 3, a score comparable to that of the dexamethasone group. We then analyzed the serum levels of IgE and histamine, which are standard markers of the early inflammatory response and involve the activation of mast cells, B cells, or monocytes. DfE application significantly elevated the levels of both markers compared with those in the control group, but these increases were significantly reduced by RA (1 or 3 mg/kg) or dexamethasone administration (Figure 1c). Additionally, the DfE-induced increase in the levels of inflammatory chemokines (RANTES, TACR, MDC, and TSLP) was also significantly decreased by RA (1 or 3 mg/kg) or dexamethasone treatment (Figure 1d). The inhibitory effects of 3 mg/kg RA on these inflammatory markers were similar to those of dexamethasone. These results suggest that RA may have an inhibitory effect on DfE-induced skin inflammation by reducing the levels of inflammatory factors.

### 2.2. RA Alleviates Histological Damage in DfE-Induced Skin Lesions

**Figure 2:**
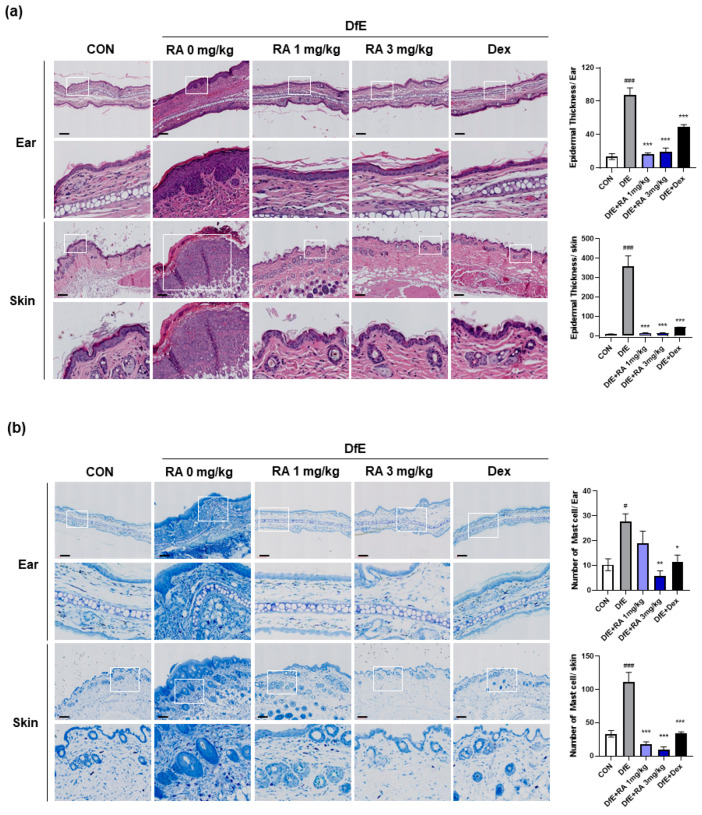
RA mitigated DfE-induced pathological features and mast cell infiltration into the skin and ear lesions of the mice. Representative images of (**a**) H&E staining and (**b**) TB staining of skin and ear tissue sections. Epidermal thickness and mast cell numbers were analyzed in the mouse skin at 40× magnification (upper panel) and 400× magnification (lower panel) via microscopy, as shown in the accompanying bar graph on the left. The data are presented as the means ± SEMs (n = 5). # *p* < 0.05 and ### *p* < 0.001 vs. the normal control group (CON); * *p* < 0.05, ** *p* < 0.01, and *** *p* < 0.001 vs. DfE. Scale bar = 100 μm.

To confirm the physiological effect of RA-mediated suppression of DfE-induced skin inflammation, we assessed skin tissue histology via hematoxylin and eosin (H&E) staining and evaluated immune cell infiltration via toluidine blue (TB) staining. Mast cell infiltration induced by proinflammatory factors in resident skin cells is characteristic of the early phase of the systemic immune response. DfE application resulted in epidermal hyperplasia, as evidenced by increased epidermal thickness in ear and skin tissues (Figure 2a). However, RA (1 or 3 mg/kg) and dexamethasone significantly and markedly reduced the thickness of the skin lesions; the epidermal thickness of the RA treatment group was similar to that of the normal control group. RA also significantly inhibited mast cell infiltration in ear and skin lesions, reaching the level of the control group (Figure 2b). Notably, at a high dose (3 mg/kg), RA was even more effective than dexamethasone (1 mg/kg) in reducing mast cell infiltration in ear and skin lesions.

### 2.3. RA Suppresses the IFN-γ/TNF-α-Induced Increase in Chemokine Levels in Keratinocytes

**Figure 3:**
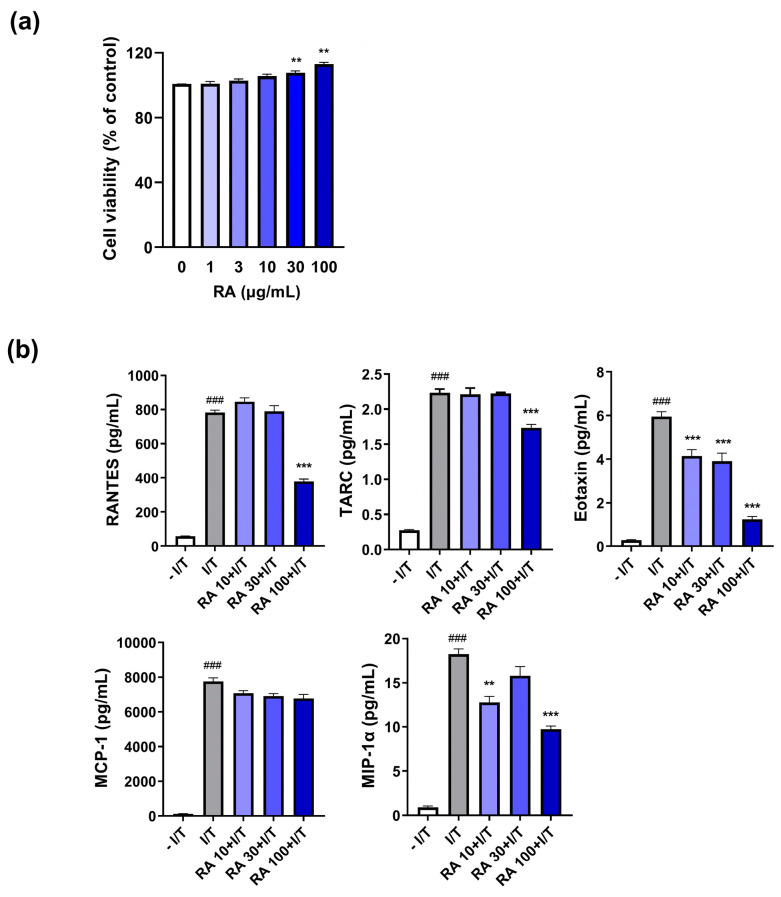
RA attenuated inflammatory-related chemokine levels in IFN-γ/TNF-α-stimulated HaCaT cells. (**a**) The cells were treated with RA (1, 3, 10, 30, or 100 µg/mL) for 24 h, and cell viability was measured by an CCK-8 assay. (**b**) The levels of five inflammatory chemokines (RANTES, TARC, eotaxin, MCP-1, and MIP-1α) in the media were measured via ELISA as described in the Section 4. ### *p* < 0.001 vs. the vehicle-treated cells without IFN-γ/TNF-α (– I/T) (10 ng/mL); ** *p* < 0.01 and *** *p* < 0.001 vs. the vehicle-treated cells with IFN-γ/TNF-α (I/T).

Inflammatory allergens stimulate keratinocytes to produce inflammatory chemokines that evoke a systemic immune response. Given that RA inhibits the serum levels of inflammatory chemokines, reflecting its inhibitory effect on the DfE-induced systemic immune response, we examined whether RA could also suppress the production of inflammatory chemokines from keratinocytes to inhibit the initiation of skin inflammation. RA at a high dose did not induce any cellular cytotoxicity in HaCaT cells (Figure 3a). IFN-γ/TNF-α increased the levels of five chemokines (RANTES, TARC, eotaxin, MCP-1, and MIP-1α) related to skin inflammation in HaCaT cells (Figure 3b). However, 100 µg/mL RA significantly decreased the levels of RANTES, TARC, eotaxin, and MIP-1α but not that of MCP-1 (Figure 3b).

### 2.4. RA Attenuates Concanavalin A (Con A)-Induced Th Cytokine Levels in Splenocytes

**Figure 4:**
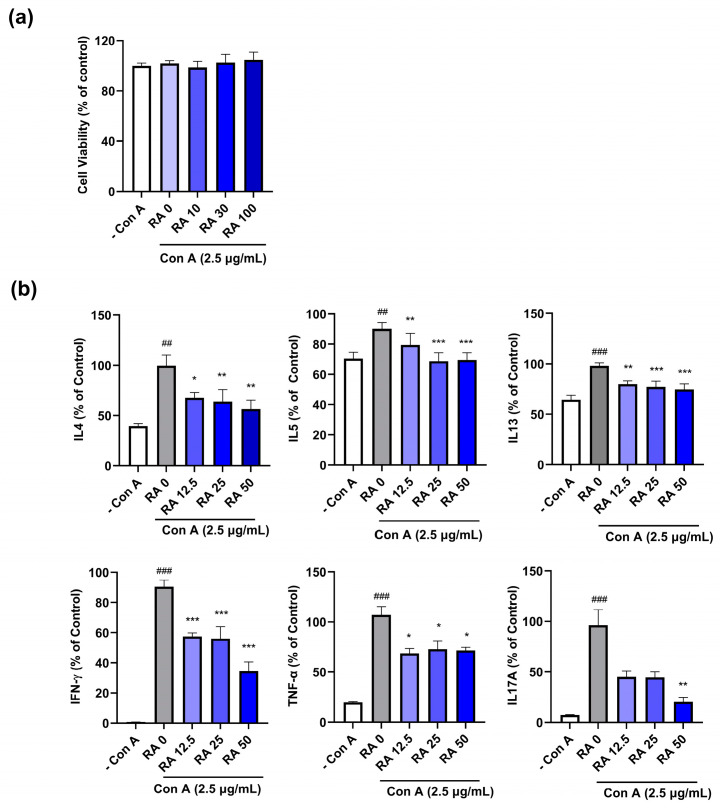
RA suppressed the Con A-induced increase in inflammatory Th cytokine levels in primary mouse splenocytes. (**a**) The viability of the splenocytes was examined after 24 h of incubation with Con A (2.5 µg/mL) or RA (0, 10, 30, or 100 µg/mL); (**b**) The levels of nine cytokines (IL-4, IL-5, IL-13, IFN-γ, TNF-α, and IL-17A) in the culture media of splenocytes were analyzed via ELISA as described in the Section 4. ## *p* < 0.01 and ### *p* < 0.001 vs. the vehicle-treated cells without Con A (– Con A); * *p* < 0.05, ** *p* < 0.01, and *** *p* < 0.001 vs. the vehicle-treated cells with Con A (RA 0).

Skin inflammatory chemokines and activated immune cells, such as mast cells, induce a systemic immune response by activating Th1/2 cells. Th2 inflammation is dominant at the initial or acute stage of skin inflammation in diseases such as AD, followed by a Th1/2 mixed response at the chronic stage [5]. Th17 cell activation also increases with increasing severity of inflammation in AD patients [33]. Thus, we examined the inhibitory effect of RA on the Con A-induced increase in Th1/2/17 cytokine levels in primary mouse splenocytes. Con A (2.5 µg/mL) and RA (0, 10, 30, or 100 µg/mL) did not induce cell cytotoxicity (Figure 4a). We found that Con A significantly induced Th1 (IFN-γ and TNF-α), Th2 (IL-4, IL-5, and IL-13), and Th17A cytokines, which were inhibited by RA treatment in primary splenocytes (Figure 4b).

### 2.5. RA Increases Nrf2/HO-1 Expression

**Figure 5:**
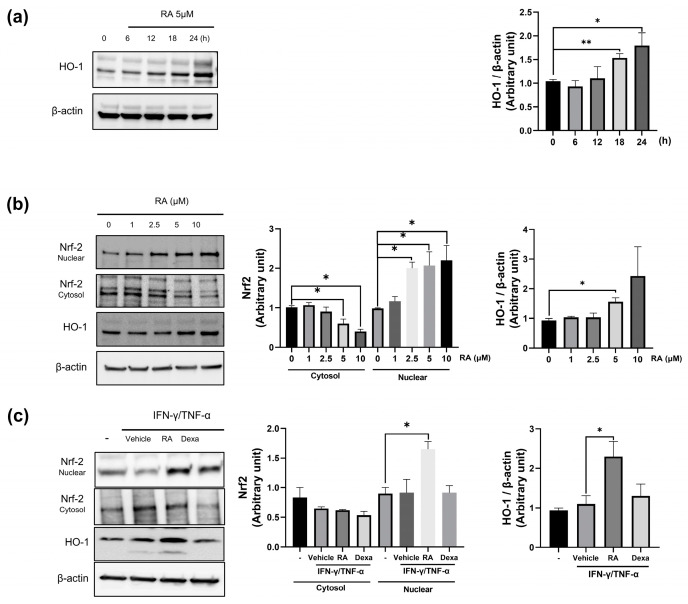
RA-induced Nrf2 and HO-1. (**a**) RA increases HO-1 expression in keratinocytes in a time-dependent manner; (**b**) RA increases cytosolic HO-1 and nuclear Nrf2 levels but decreases cytosolic Nrf2 levels in a dose-dependent manner. HO-1 and Nrf2 levels were quantified by densitometric analysis. Nrf2 and HO-1 levels were examined in keratinocytes after RA (1, 2.5, 5, or 10 μM) treatment; (**c**) RA increases nuclear Nrf2 and cytosolic HO-1 levels in cells treated with IFN-γ/TNF-α (I/T) (10 ng/mL). Vehicle: DMSO-treated cells; RA: RA (5 μM)-treated cells; Dexa: dexamethasone (20 μM)-treated cells. The quantified Western blot data are shown as the means ± standard deviations (SDs) (*n* = 3). * *p* < 0.05, and ** *p* < 0.01 vs. the vehicle-treated cells as determined by two-tailed t tests.

To explore the molecular mechanism by which RA protects against skin inflammation, we focused on the Nrf-2/HO-1 pathway. Our results revealed that RA induces HO-1 expression in a time-dependent manner, with HO-1 expression peaking after 24 h of incubation with RA (Figure 5a). While RA gradually decreased cytosolic Nrf-2 levels, it significantly increased nuclear Nrf-2 expression in a dose-dependent manner (Figure 5b). Correspondingly, high doses of RA (5 and 10 μM) increased HO-1 expression. The increase in nuclear expression after RA treatment, coupled with the reduction in cytosolic Nrf2 expression, may suggest that Nrf2 is translocated to the nucleus to upregulate HO-1 expression. Notably, 5 μM RA significantly increased nuclear Nrf-2 expression and further increased cytosolic HO-1 expression in IFN-γ/TNF-α-treated keratinocytes (Figure 5c). IFN-γ/TNF-α treatment appeared to reduce cytosolic Nrf-2 levels, although the difference was not statistically significant.

## 3. Discussion

In this study, we investigated the inhibitory effect of RA on mice with AD-like skin inflammation induced by repeated topical application of DfE. RA was found to activate the Nrf-2/HO-1 pathway to alleviate ROS-induced oxidative stress in keratinocytes, which could contribute to the inhibition of DfE-induced local and systemic inflammatory reactions in the skin.

Chemical or biological allergens initially induce skin inflammation by recruiting mast cells or dendritic cells to activate the T-cell immune response. Repeated DfE application on NC/Nga mice spontaneously induces excessive oxidative stress to induce redox alterations in skin lesions [3]. It initiates mast cell accumulation and a T-cell-mediated immune response, increasing the levels of Th1/2 cytokines in skin lesions of NC/Nga mice [34], which mirrors the early phase immune response of skin inflammation observed in human AD patients. Previous studies have suggested that the pharmaceutical activity of RA against AD-like skin inflammation involves regulating the initial process of the immune response [35,36]. Subcutaneous injection of RA into the dorsal skin of mice alleviates dibutyl square acid (SADBE)-induced pruritus and skin inflammation by inhibiting the MRGPRX2-PLCγ1PKC-NF-κB axis, which is involved in mast cell activation [35]. Additionally, the intraperitoneal injection of RA inhibits DNFB-induced CD4+ and CD8+ T-cell infiltration in mouse skin lesions and decreases Th1/2 cytokine levels in CD4+ T-cells [19]. Consistent with these previous studies, we found that oral administration of RA inhibited DfE-induced increases in the levels of inflammatory chemokines (RANTES, TARC, MDC, and TSLP) in the serum and mast cell infiltration in skin lesions. These chemokines primarily function to induce the infiltration of eosinophils and mast cells in skin lesions during the early phase of inflammation, contributing to the activation of T-cells [5,37]. Repeated topical application of DfE increases the level of ROS in the dermis [3], resulting in the excessive accumulation of mast cells and a T-cell-mediated immune response, increasing the levels of Th1/2 cytokines in NC/Nga mice [34]. Thus, our results suggest that RA reduces DfE-induced inflammatory factor levels, including ROS, thereby decreasing mast cell infiltration in DfE-induced AD-like model mice at the early disease phase.

Keratinocytes produce chemokines and Th2 cytokines, including interleukin (IL)-4 and IL-13, to downregulate skin barrier protein expression in keratinocytes and stimulate the apoptosis of keratinocytes, contributing to cutaneous spongiosis in acute skin lesions [38,39]. Inflammatory cytokines or chemokines stimulate the initiation of systemic CD4+ T-cell activation in the lymph nodes or spleens of DfE-induced AD model mice [40,41]. Increased levels of Th2 cytokines reflect a predominant Th2 response at the acute stage of AD, while it becomes a Th1/Th2 mixed response at chronic stages, resembling the pathological phenotypes of human AD [5,42]. Additionally, keratinocyte-derived chemokines (e.g., thymic stromal lymphopoietin) enhance the itching sensation, which can lead to increased scratching and worsening of skin lesions [43]. In this study, we also reported that RA inhibited Con A-induced Th1/Th2/Th17 inflammatory cytokine levels in mouse splenocytes and inhibited the levels of IFN-γ/TNF-α-induced chemokines (RANTES, TARC, eotaxin, and MIP-1α) in keratinocytes. Several herbal components or medicinal herbs inhibit DfE-induced AD-like skin inflammation by attenuating immune cell infiltration, alleviating skin barrier dysfunction, or mitigating the Th1/2 imbalance [44,45]. Therefore, the inhibitory effects of RA on chemokine levels in keratinocytes and on Th cytokine generation in splenocytes could contribute to alleviating immune cell infiltration and systemic T-cell activation, subsequently reducing DfE-induced skin inflammation in mouse skin lesions.

The Keap1–Nrf2 pathway is a crucial cytoprotective mechanism that transcriptionally regulates antioxidant response elements (AREs) of antioxidant proteins, such as HO-1, in response to oxidative and electrophilic stress [46]. Various phytochemicals or small molecules indirectly modulate the Keap1–Nrf2 pathway by forming covalent adducts with the sulfhydryl groups of cysteine or through electrophilic reactions with cysteine in Keap1 [47]. This interaction has dual effects: it prevents the association of Keap1 with Cullin for ubiquitin-mediated degradation of Nrf2 and induces allosteric changes in Nrf2-interacting sites in Keap1, releasing Nrf2 for nuclear translocation [47,48]. RA modulates the Keap1–Nrf2 pathway via covalent modification of the Michael acceptor of RA with the cysteine 151 residue in the N-terminal BTB (Broad complex, Tramtrack, and Bric-a-Brac) domain of Keap1 in inflammation-stimulated RW264.7 cells [49]. When cysteine residues of the BTB and IVR domains in Keap1 are covalently modified under oxidative stress, homodimerization of Keap1 and Cullin for Nrf2 degradation is disturbed, resulting in the release of Nrf2 for translocation [48]. Additionally, RA can interact with both polar and hydrophobic residues in the Kelch 1-6 domains of Keap1, which consist of positively charged P1 and P2 subpockets and hydrophobic P4 and P5 subpockets for Nrf2 binding [50,51]. The electrostatic and hydrophobic interactions between RA and Keap1 could decrease the binding strength of Keap1 that results in the dissociation of Keap1 from Nrf2 [52]. In keratinocytes, RA exhibits excellent antioxidant properties by abrogating UVB-induced reduction in Nrf2 expression and increasing the expression of antioxidant proteins such as glutamate-cysteine ligase catalytic subunit (GCLC) and glutathione synthetase (GSS) to protect the skin from UVB-induced oxidative damage [22,53]. In this study, we found that RA increased the nuclear translocation of Nrf2 to induce HO-1 expression in keratinocytes in a dose- and time-dependent manner. RA profoundly increased Nrf2/HO-1 expression in IFN-γ/TNF-α-treated keratinocytes. These findings suggest that the interaction of RA with Nrf2 regulatory domains of Keap1 could induce allosteric changes in Keap1 to disassemble the Keap1–Nrf2 complex, leading to the release and nuclear translocation of Nrf2, which consequently increases HO-1 expression to protect against oxidative stress in keratinocytes.

RA inhibits skin inflammation by regulating various molecular pathways. RA inhibits imiquimod-induced psoriasis-like dermatitis in mice by reducing JAK2/Stat3-dependent Th17 cell differentiation and IL-17A production [54]. RA also significantly suppresses the poly(I:C)-induced expression of inflammatory cytokines, including Il-1β and TNF-α, in human keratinocytes by suppressing the NF-κB pathway [55]. We also found that RA inhibited DfE-induced skin inflammation by attenuating chemokine and Th1/2/17 cytokine generation and increasing Nrf2/HO-1 expression to decrease inflammatory factor-induced oxidative stress. The topical application of RA to improve epidermal conditions in AD patients by reducing transepidermal water loss and erythema on the antecubital fossa [20] could suggest the pharmaceutical potential of RA in treating skin inflammatory diseases such as AD or psoriasis through various molecular pathways. Further studies investigating these relevant action mechanisms of RA against skin inflammation, along with the therapeutic safety of RA in clinical conditions, could be beneficial for developing skin application remedies.

Skin also functions as a neuroendocrine organ, mediating the nervous, immune, and endocrine systems in response to skin stress [56]. Chronic itching inevitably triggers scratching behavior via the IgE–mast-cell–histamine-neuronal axis [57]. The persistence of this itching–scratching cycle connecting the immune and neuronal systems exacerbates atopic inflammation and skin barrier dysfunction. Further research is needed to investigate the effects of RA on neurohormonal mediators or cytokines involved in neuroendocrine signaling within the skin. It could elucidate how RA modulates various nervous and immune pathways for the pharmaceutical activity of RA.

## 4. Materials and Methods

### 4.1. Materials

House dust mite ointment was purchased from Biostir Inc. (Kobe, Japan). The LBIS Mouse IgE ELISA Kit was purchased from Fujifilm (Shibukawa, Japan). Histamine Research ELISATM was purchased from LDN (Nordhorn, Germany). The LEGEND-plex™ Mouse Proinflammatory Chemokine Panel was purchased from BioLegend (San Diego, CA, USA). The toluidine blue staining kit was purchased from VitroVivo Biotech (Rockville, MA, USA). H&E staining kit, Roswell Park Memorial Institute (RPMI) 1640 medium, fetal bovine serum (FBS), penicillin/streptomycin, NE-PER Nuclear and Cytoplasmic Extraction Reagents, BCA assay kit, TNF-α, IFN-γ, and Super Signal West Femto Chemiluminescent Substrate were purchased from Thermo Fisher Scientific (Waltham, MA, USA). Concanavalin A was purchased from Sigma Aldrich (Burlington, MA, USA). Normal human epidermal keratinocytes (HEKs) (catalog number 00192627) and KBM-Gold Basal medium supplemented with a SingleQuots Supplement pack were purchased from LonzaBioscience (Basel, Switzerland). HaCaT human keratinocyte cells were purchased from American Type Culture Collection (Manassas, VA, USA). Cell Counting Kit-8 (CCK-8) was purchased from Dojindo Molecular Technologies Inc. (Rockville, MD, USA). Antibodies against Nrf2 (12721), HO-1 (70081), and β-actin (4967) were purchased from Cell Signaling Technology (Boston, MA, USA).

### 4.2. Animals

The animal experimental protocol was designed according to the Guide for the Care and Use of Laboratory Animals of the National Institutes of Health (2013). The protocol for the AD experiment was reviewed and approved by the Institutional Animal Care and Use Committee (IACUC) at the Korea Institute of Oriental Medicine (KIOM) (Approval No. 22-073; approval date, 1 August 2022). The animal experiment for the isolation of splenocytes to analyze Th cytokines was approved by the IACUC of KIOM (approval number: 23-021; Approval date, 30 March 2022). All methods are reported in accordance with the Animal Research: Reporting of In Vivo Experiments (ARRIVE) guidelines for the reporting of animal experiments. NC/Nga male mice (body weight 19–24 g, 8 weeks old) (Central Lab Animal Inc., Seoul, Republic of Korea) were housed in a specific-pathogen-free (SPF) facility at KIOM, where they were maintained under standard laboratory animal conditions including a controlled temperature (23 ± 2 °C) and humidity (55 ± 10%) level and a 12 h light–dark cycle. The mice were provided a standard laboratory diet, and water was provided ad libitum.

### 4.3. AD Induction and Dermatitis Scoring

The animals were randomly categorized into five groups (*n* = 5): the Nc/Nga normal control group, which was administered phosphate-buffered saline (PBS); the DfE-induced AD group administrated PBS; the DfE-induced AD group administrated 1 mg/kg RA; the DfE-induced AD group administrated 3 mg/kg RA; and the DfE-induced AD group administrated 1 mg/kg dexamethasone (positive control) [56]. All the treatments were administered orally once daily. AD-like skin lesions were induced by sensitization to 200 μL of 4% SDS sprayed on the dorsal skin and ear of the mice, which were shaven the previous day. House dust mite DfE ointment (100 mg, Biostir Inc., Kobe, Japan) was repeatedly applied on the dorsal skin and ears twice a week for 3 weeks. After 7 days of AD induction, RA (1 or 3 mg/kg) or dexamethasone (1 mg/kg) was orally administered by a syringe with plastic feeding tubes for the remaining 14 days. Ear thickness was measured via a digital caliper (CAS©, Seoul, Republic of Korea) twice a week after the health status of each mouse was assessed. Dermatitis scoring was based on the sum of individual scores for AD-related symptoms (scarring/dryness, erythema/hemorrhage, edema, and excoriation/erosion), ranging from 0 (no symptoms) to 1 (mild), 2 (moderate), or 3 (severe), as determined by the scorer [57].

### 4.4. Inflammatory Factor Analysis

After the final treatment of the sample and a 12 h fasting period, the mice were sacrificed using CO_2_ anesthesia, and the serum samples were collected. Following centrifugation at 3000 rpm for 15 min, the serum was collected and stored at −70 °C. Inflammatory factors in the serum or cell culture media were quantified via the LBIS Mouse IgE ELISA Kit (Fujifilm), Histamine Research ELISATM (LDN), or the LEGEND-plex™ Mouse Proinflammatory Chemokine Panel (BioLegend) according to the manufacturer’s instructions. A SpectraMax and BD LSRFortessa™ flow cytometer (BD Biosciences, San Jose, CA, USA) with BD CellQuest™ and LEGENDplex™ Software v8.0 (VigeneTech Inc., Carlisle, MA, USA) were used for the experiments.

### 4.5. Histological Analysis

The excised tissues were preserved in a 10% formaldehyde solution and then embedded in paraffin for cryosectioning. H&E staining was performed to evaluate hyperkeratosis and epidermal hyperplasia of the skin tissue. To identify mast cell infiltration, a toluidine blue staining kit (VitroVivo Biotech) was employed. A digital slide scanning system (Kfbio, Ningbo, China) in semiautomatic mode was used to scan and analyze the stained tissue samples.

### 4.6. Cell Culture and Viability Assay

The spleens of BALB/c mice (Central Lab Animal Inc., Seoul, Republic of Korea) were mashed with a medical spatula and filtered into a culture dish using a 70 μm cell strainer. After removing red blood cells (RBCs) via incubation with an RBC lysis buffer for 5 min, the cells were collected using centrifugation. The isolated primary splenocytes were incubated in RPMI 1640 medium with 10% FBS and 1% penicillin/streptomycin (Thermo Fisher Scientific) [58,59]. Normal human epidermal keratinocytes (HEKs) (catalog number 00192627, LonzaBioscience) were cultured in KBM-Gold Basal medium supplemented with a SingleQuots Supplement Pack (LonzaBioscience). HaCaT human keratinocyte cells (American Type Culture Collection) were cultured in Dulbecco’s modified Eagle’s medium supplemented with heat-inactivated 10% FBS and the above antibiotics in a Heracell^TM^ incubator (Thermo Fisher Scientific) with 5% CO_2_ at 37 °C. TNF-α and IFN-γ (10 ng/mL) (Thermo Fisher Scientific) were used to activate the keratinocytes, and Con A (2.5 μg/mL) (Sigma Aldrich) was used to treat the splenocytes in the culture medium.

The cells were incubated in growth media supplemented with various concentrations of RA (0–100 µg/mL) for 24 h. After adding 10% CCK-8 (Dojindo Molecular Technologies Inc.) reagent to the media, the colorimetric results of the viability assay were measured at a wavelength of 450 nm using a SpectraMax 340 microplate reader (Molecular Devices, San Jose, CA, USA) according to the manufacturer’s instructions.

### 4.7. Western Blot Analysis

Detergent-based whole-cell lysis with RIPA buffer (Thermo Fisher Scientific) was performed to extract total cellular protein. NE-PER Nuclear and Cytoplasmic Extraction Reagents (Thermo Fisher Scientific) were used for fractionation. A BCA assay was performed to quantify the protein concentration (Pierce™ BCA assay kit, Thermo Fisher Scientific). The extracted proteins were separated by their molecular weight under denaturing conditions with SDS on Mini-PROTEAN TGX precast protein gels (Bio-Rad, Hercules, CA, USA). The separated proteins were then electrically transferred to polyvinylidene fluoride membranes via a Trans-Blot Turbo Transfer system (Bio-Rad) under semidry conditions. The membranes were blocked with a commercial blocking solution (Atto, Tokyo, Japan) for 1 h and subsequently incubated with specific primary or HRP-conjugated secondary antibodies for an additional recommended time. Visualization of the immunoreactive bands was achieved via the Super Signal West Femto Chemiluminescent Substrate (Thermo Fisher Scientific) with a computerized densitometer (Bio-Rad).

### 4.8. Statistical Analysis

The data are presented as the means ± SEMs. A one-way ANOVA followed by Dunnett’s test was employed for multiple group comparisons to verify statistical significance. GraphPad Prism software (Version 8.0; GraphPad Software, Inc., San Diego, CA, USA) was used for the analysis. *p* < 0.05 was considered to indicate statistical significance.

## 5. Conclusions

In conclusion, this study demonstrated that oral administration of RA effectively alleviated DfE-induced AD-like skin lesions in a mouse model of AD by decreasing the levels of inflammatory cytokines and chemokines. Additionally, RA promotes HO-1 activation in keratinocytes by inducing Nrf2 expression, which may contribute to reducing the DfE-induced oxidative stress that triggers skin inflammation. These findings underscore the anti-inflammatory and antioxidant properties of RA against DfE-induced AD-like skin conditions, suggesting its potential as a pharmaceutical agent for preventing skin inflammation-related diseases such as AD.

## Data Availability

All collected data are presented in this article.
